# Next-Generation Dental Care: Robotics Shaping the Future of Dentistry

**DOI:** 10.7759/cureus.93817

**Published:** 2025-10-04

**Authors:** Rawan S Alrehaili, Ali Alshuhaib, Ridha Alali, Mohammed Alnemer, Zainab Almubarak, Majid Alsafwani, Faisal Alhalal, Ahmed Almousa, Waseem Alharthi, Suliman Alshammari, Ibrahim Alquwaie, Nouf Aldahri, Sabreen Hamdi, Ali Addokhi

**Affiliations:** 1 Dentistry, Private Sector, Medina, SAU; 2 College of Dentistry, Imam Abdulrahman Bin Faisal University, Dammam, SAU; 3 Faculty of Dentistry, King Abdulaziz University, Jeddah, SAU; 4 College of Dentistry, Jazan University, Jazan, SAU; 5 Dental Services, Eastern Health Cluster, Dammam, SAU

**Keywords:** computer-guided dentistry, dental robotics, digital dentistry, precision dentistry, robot-assisted surgery

## Abstract

Robotics has moved beyond the experimental stage and is now beginning to influence clinical practice in dentistry by setting higher standards for precision, efficiency, and personalized care. Recognizing its potential to reduce variability, enhance treatment outcomes, and reshape clinical workflows makes it essential to evaluate its current role and trajectory. This narrative review explores the historical evolution of dental robotics from early mechanical devices to advanced systems, three-dimensional imaging, and real-time navigation. It provides a cross-specialty analysis of robotic applications and highlights key technologies. Despite the promise of improved clinical outcomes and workflow standardization, the widespread adoption of robotics in dentistry faces notable challenges, including high costs, integration difficulties, training requirements, and regulatory uncertainty. Future work is likely to focus on better decision-support software and on ways to extend access through remotely guided procedures. As innovation continues, robotics may become a cornerstone of digital dentistry, but its widespread impact will depend on overcoming cost barriers and integration challenges.

## Introduction and background

The field of dentistry is undergoing a profound transformation driven by rapid advancements in digital technologies. Among these, robotics has emerged as a disruptive innovation that promises to redefine the standards of precision, efficiency, and personalization in dental care [1]. Initially introduced in medical domains such as general surgery, neurosurgery, and orthopedics, robotic systems are now making significant inroads into various dental specialties. The anatomical complexity of the oral cavity, characterized by limited access, dynamic soft tissues, and the need for micrometer-level precision, presents unique challenges that make dental procedures particularly suitable for robotic assistance [2]. Robotics in dentistry spans a wide spectrum, from haptic simulators used in dental education to AI-assisted systems capable of autonomous implant placement and real-time surgical navigation [3]. This integration offers considerable advantages, including enhanced procedural accuracy, reduced operator variability, improved ergonomics, and data-driven treatment planning [4].

Concurrently, the development of technologies such as cone beam computed tomography (CBCT), computer-aided design and manufacturing (CAD/CAM), machine learning, and real-time imaging has created a fertile ecosystem for robotics to flourish [5]. These technologies have enabled robotic platforms to evolve from static mechanical devices to intelligent, adaptable systems capable of making intraoperative decisions and providing interactive feedback control. Robotics now plays a central role in digital dentistry. What once served as peripheral support has become an integral part of daily clinical routines. Despite its promise, the adoption of robotics in dentistry remains tempered by significant challenges. These include high system costs, steep learning curves, integration barriers within clinical workflows, and unresolved regulatory and ethical concerns [6]. Nonetheless, the momentum in research and commercial development suggests that dental robotics is transitioning from experimental innovation to clinical reality [7,8]. Given the rapid pace of innovation and the diverse range of robotic systems emerging across dental specialties, there is a growing need for a comprehensive narrative review that synthesizes these developments in a clinically meaningful way. Most existing literature focuses narrowly on specific applications or technologies, often without providing context on how these systems function within the broader landscape of dental care. A narrative approach allows for the integration of technical, clinical, and educational perspectives while identifying overarching trends, limitations, and opportunities for future advancement.

Therefore, this narrative review explores the historical evolution, current applications, technological infrastructure, and future directions of robotics in dentistry. It aims to provide a cross-specialty perspective that highlights both the transformative potential and the practical considerations associated with integrating robotic systems into modern clinical practice.

## Review

Search strategy

A structured literature search was conducted across three major databases - PubMed, Scopus, and Web of Science - to identify relevant publications on robotics in dentistry. The search spanned studies published from January 2013 to August 2025, with a focus on recent advancements in robotic technologies applied across multiple dental specialties. The search terms combined controlled vocabulary and free-text words using Boolean operators. A representative search string included: ("dental robotics" OR "robot-assisted dentistry" OR "robotic systems in dentistry") AND ("endodontics" OR "orthodontics" OR "oral and maxillofacial surgery" OR "implant dentistry" OR "AI in dentistry") AND ("robot" OR "automation" OR "artificial intelligence")* Search filters were applied to limit results to studies published in English and in peer-reviewed journals. Duplicates were removed, and titles and abstracts were screened for eligibility.

Inclusion and Exclusion Criteria

Articles were considered eligible if they described the application or development of robotic systems in any field of dentistry, including specialties such as endodontics, orthodontics, pediatric dentistry, implant dentistry, and oral and maxillofacial surgery (OMFS). Studies were also included when they reported on technologies related to automation, robotic-assisted procedures, AI, or haptic feedback systems. Eligible papers could take the form of technical reports, clinical investigations, experimental studies, or conceptual discussions, provided they were published in English and accessible in full text. Although AI increasingly underpins many digital tools in dentistry, this review focuses specifically on robotics, while acknowledging areas where AI functions integrate into robotic systems, such as real-time navigation and treatment planning. In contrast, studies were excluded if they dealt exclusively with general medical robotics without a dental context, lacked relevance to dental practice, or had not undergone peer review.

Evolution of robotics in dentistry

The evolution of robotics in dentistry has mirrored the broader trajectory of medical robotics, shaped by rapid advances in computing, imaging, and engineering. However, its development has been uniquely influenced by the anatomical and procedural complexities of the oral cavity, which demand exceptional precision within a constrained and dynamic environment. Over the last two decades, dental robotics has progressed from rudimentary mechanical devices and simulation tools to intelligent, integrated systems capable of real-time navigation and adaptive decision-making. Early robotic technologies in dentistry were primarily confined to educational and research settings. These systems were designed to simulate jaw movement, model occlusal patterns, or deliver repetitive motion for training purposes. While valuable in controlled environments, they lacked the functional sophistication and clinical applicability needed for direct patient care. Their utility was largely limited by insufficient sensor resolution, slow data processing, and inadequate integration with diagnostic imaging [9].

The turning point in the evolution of dental robotics came with the convergence of several technological innovations: digital radiography, three-dimensional (3D) imaging (especially CBCT), intraoral scanning, advanced sensors, and real-time tracking algorithms. These breakthroughs enabled the design of more agile and responsive robotic systems capable of translating digital treatment plans into precise physical actions with submillimeter accuracy. Another key milestone in the field was the development of computer-aided design and manufacturing (CAD/CAM) technologies, which laid the foundation for customized, data-driven workflows. Robotics began to move beyond simulation into clinical implementation, as systems were developed to automate repetitive, high-precision tasks while interfacing with digital planning tools. This marked a shift from operator-dependent procedures toward greater reproducibility, efficiency, and outcome predictability [10].

Recent developments have pushed dental robotics even further into the realm of intelligent automation. Modern systems increasingly incorporate artificial intelligence (AI), machine learning, and haptic feedback, enabling them to respond dynamically to intraoperative variables. Some platforms now support closed-loop feedback, adjusting operational parameters in real time based on force detection, tissue resistance, or positional deviation. These advancements reflect a broader trend from static assistance to cognitive augmentation of clinical practice.

Today, robotics in dentistry is no longer seen as a futuristic concept but as an evolving reality. It aligns closely with the goals of digital dentistry, which are to enhance accuracy, reduce treatment variability, and provide a more personalized patient experience. As robotics continues to evolve alongside data science, materials engineering, and human-machine interface technologies, it is poised to become a foundational element in the next generation of dental care [11]. Table 1 depicts major milestones in the historical development of robotics in dentistry.

**Table 1:** Milestones in the history and evolution of robotics in dentistry. CAD/CAM, computer-aided design/computer-aided manufacturing; CT, computed tomography; CBCT, cone beam computed tomography; AI, artificial intelligence; 3D, three-dimensional

Period	Milestone/development	Technological enabler	Impact on dentistry
Pre-2000	Introduction of mechanical simulators (e.g., jaw movement)	Kinematics, motors	Educational use; no clinical application
Early 2000s	Adoption of CAD/CAM and CBCT in digital planning	3D imaging, digital modeling	Enabled virtual treatment planning
Mid-2000s	Development of static guidance and simulation-based robotic systems	CT/CBCT, optical scanning	Initiated semi-automated interventions
2010-2015	Robotic assistance in surgical and restorative tasks	Haptic feedback, AI algorithms	Improved accuracy and repeatability
2015-present	AI-integrated, real-time robotic platforms	Deep learning, cloud computing	Emergence of intelligent, adaptive robotic systems

Classification of dental robotics

The classification of robotics in dentistry can be approached through multiple lenses, including the degree of autonomy, functional role, clinical application, and integration with digital systems. As robotic technologies have matured, a clearer taxonomy has emerged, facilitating understanding of their capabilities and limitations within various dental specialties [12].

Degree of Autonomy

Dental robotics can be broadly categorized into three levels of autonomy: assistive, semi-autonomous, and autonomous systems. From the perspective of autonomy, robotic platforms range from assistive to semi-autonomous and fully autonomous. Assistive robots are entirely controlled by the clinician and serve primarily to enhance stability, magnification, or ergonomic support. Semi-autonomous robots, such as the Yomi system, operate in collaboration with the operator, executing tasks with haptic feedback and trajectory control while still allowing real-time human intervention. Although autonomous robots are not yet widely used in clinical practice, they are being developed in research settings, with experimental systems capable of performing endodontic or diagnostic tasks without direct human manipulation [13].

Clinical Function

Robotic systems in dentistry may also be classified by their primary function. When classified by function, robotic systems may be surgical, therapeutic, diagnostic, or educational in nature. Surgical robots are intended to support invasive procedures, including implant placement, bone cutting, or endoscopic navigation during maxillofacial surgery. Therapeutic robots, by contrast, assist in non-surgical interventions such as automated wire bending in orthodontics or experimental robotic canal preparation tools in endodontics. Diagnostic and imaging robots contribute by improving the accuracy of radiographic or scanning procedures, for example, through automated patient positioning during CBCT acquisition. Finally, educational and simulative robotics play an essential role in training, with haptic simulators that replicate tactile resistance and provide realistic practice opportunities for dental students [14].

Integration With Digital Systems

**Figure 1:**
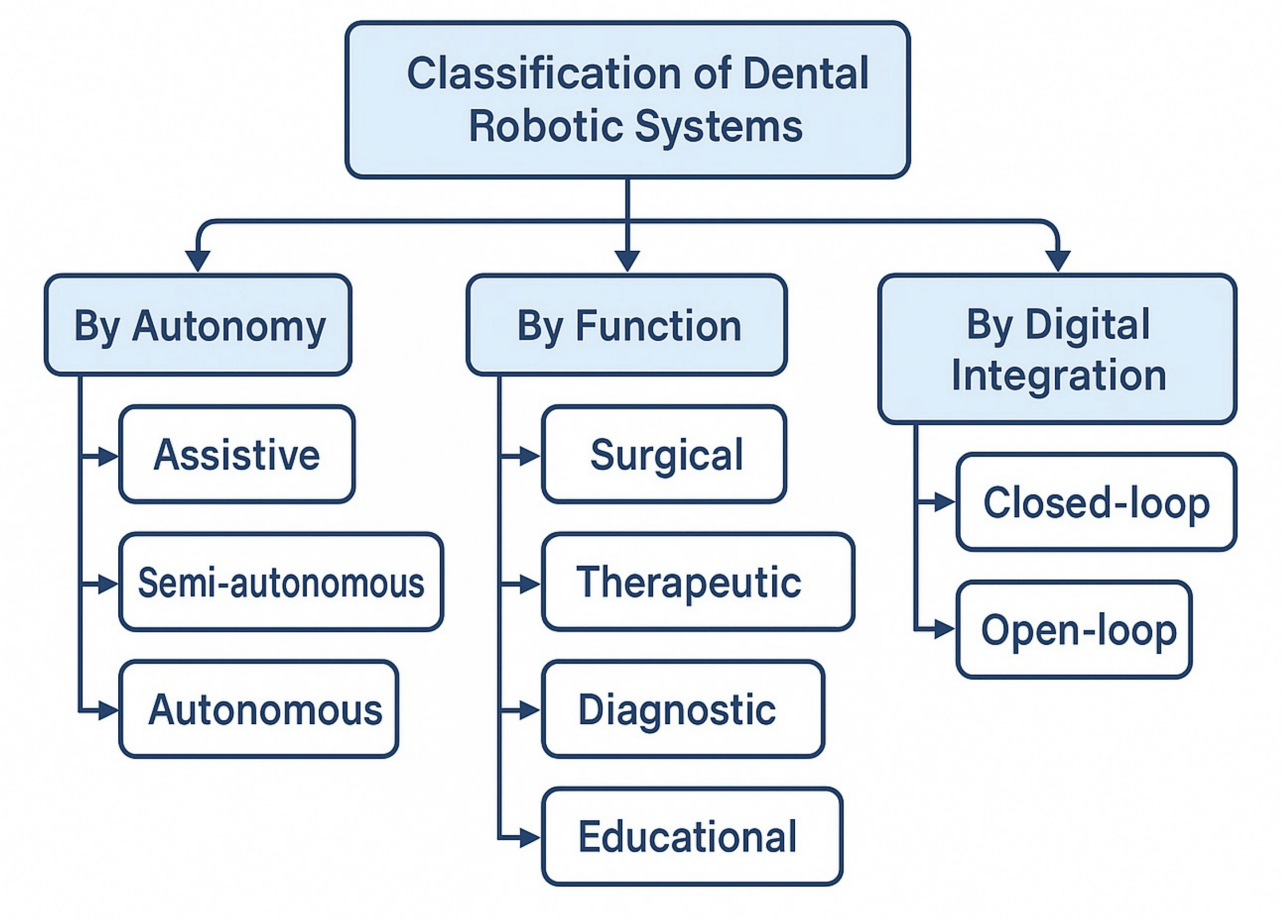
Classification of dental robotics based on autonomy, function, and digital integration Image credit: Rawan S. Alrehaili.

Modern dental robotics often functions as a component within broader digital dentistry ecosystems. Integration with AI algorithms, CAD/CAM platforms, and virtual treatment planning tools allows for streamlined workflows from diagnosis through to treatment delivery. Closed-loop robotic systems are able to adjust their performance in real time by responding to sensory feedback, thereby increasing accuracy and safety. Open-loop systems, in contrast, follow pre-programmed instructions without intraoperative correction and are therefore most suited to tasks with low variability [15]. A schematic overview of dental robotic systems is shown in Figure 1.

Robotics in endodontics

Robotic Systems For Endodontic Training

A fundamental application of robotics in endodontics lies in preclinical training and simulation. Haptic-enabled dental simulators, such as SIMROID, offer students a platform to practice root canal procedures in a virtual environment that replicates clinical scenarios [16]. These systems are equipped with force sensors and motion tracking capabilities that simulate the tactile resistance encountered during instrumentation. Learners receive immediate feedback on depth control, force magnitude, and angulation, allowing for iterative improvement. Preliminary studies on haptic-enabled simulators suggest potential benefits for enhancing spatial awareness and reducing certain procedural errors; however, the evidence remains limited and context-dependent [17,18].

Micro-robots in Root Canal Procedures

The concept of microrobotic devices for intra-canal operations has been proposed as a potential future advancement in endodontic robotics. These miniature robots, envisioned to be less than a few millimeters in diameter, are being explored for their ability to navigate narrow and curved root canal systems. While early experimental studies have suggested possible approaches for debris removal and irrigant delivery, fully functional prototypes demonstrating reliable intra-canal performance are not yet available [19]. Experimental concepts include microrobotic end-effectors with magnetic actuation for precise manipulation in the confined root canal space, as well as catalytic micro-motors designed to disrupt biofilms and enhance cleaning efficacy [19,20]. These approaches are largely exploratory and often envisioned to rely on CBCT-derived data for navigation, potentially transforming static imaging into dynamic operative planning [20]. While clinical application remains in the experimental phase, the potential of these microrobots to improve access in anatomically challenging cases, such as calcified canals or complex curvatures, is noteworthy. Their ability to perform mechanical tasks with consistent force and direction could minimize procedural mishaps such as ledging, transportation, or instrument separation [20].

Automated Instrumentation and Navigation

The application of robotics to canal instrumentation is largely conceptual and under experimental development. Proposed systems aim to integrate robotic motion control with digital imaging and AI-based decision support, using CBCT data to generate virtual treatment paths and potentially incorporating real-time positional tracking, torque monitoring, and pressure sensing [14]. While these innovations remain exploratory, current clinical practice relies on operative microscopes, which play a central role in enhancing visualization, precision, and safety during instrumentation and irrigation. Microscopes significantly improve the identification of canal orifices, detection of microcracks, and control of irrigant delivery, thereby reducing the risk of complications such as apical extrusion. Future robotic approaches are likely to build upon and complement the established advantages of the dental operating microscope rather than replace it.

Diagnostic and Imaging Integration

Exploratory approaches have proposed combining robotics with advanced imaging tools to improve diagnostic precision in endodontics. For instance, robotic integration with intraoral scanners or fiber-optic microendoscopes could theoretically enhance visualization of complex canal anatomy, including lateral canals, isthmuses, and apical deltas, which may not always be detected on conventional radiographs. Optical coherence tomography (OCT) has been investigated in dental applications, but no clinically validated intraoral robotic OCT system currently exists. Preliminary ex vivo studies using handheld or benchtop OCT units have shown feasibility in visualizing root canal microstructures, though these remain research-stage technologies. At present, robotic-AI integration in endodontic imaging is conceptual: AI algorithms are being developed for canal morphology classification and treatment planning, but their incorporation into robotic scanning platforms is yet to be realized [21].

Robotics in Guided Endodontic Surgery

Robotic technology is also being explored in the context of endodontic microsurgery, particularly in procedures such as apicoectomy or root-end resection. These interventions require exceptional spatial precision, especially when performed near vital anatomical structures such as the maxillary sinus or inferior alveolar nerve. A recent case report described the use of the assisted targeting robot (ATR) system in apicoectomy, integrating CBCT-based planning with robotic navigation to guide the operator along a predefined osteotomy path with submillimeter accuracy during removal of a fractured file beyond the apical foramen [22]. While this demonstrates the technical feasibility and potential for enhanced precision, it remains a single case report and therefore cannot validate the broader clinical efficacy of the method; further controlled studies are needed to establish its reliability and outcomes. Robotic-assisted surgical platforms can integrate CBCT data to support 3D planning and guide osteotomy execution. Conceptually, the addition of dynamic navigation allows for real-time adjustment during procedures, which may help compensate for patient movement and anatomical variability. These developments aim to enhance surgical accuracy and reduce intraoperative trauma, although current evidence remains preliminary [23]. Some pilot studies have demonstrated that robotic systems can reduce deviations in osteotomy paths to less than 0.5 mm, enhancing the likelihood of successful root-end access and retrograde filling [24,25].

Robotics in Retreatment and Post-Removal

**Figure 2:**
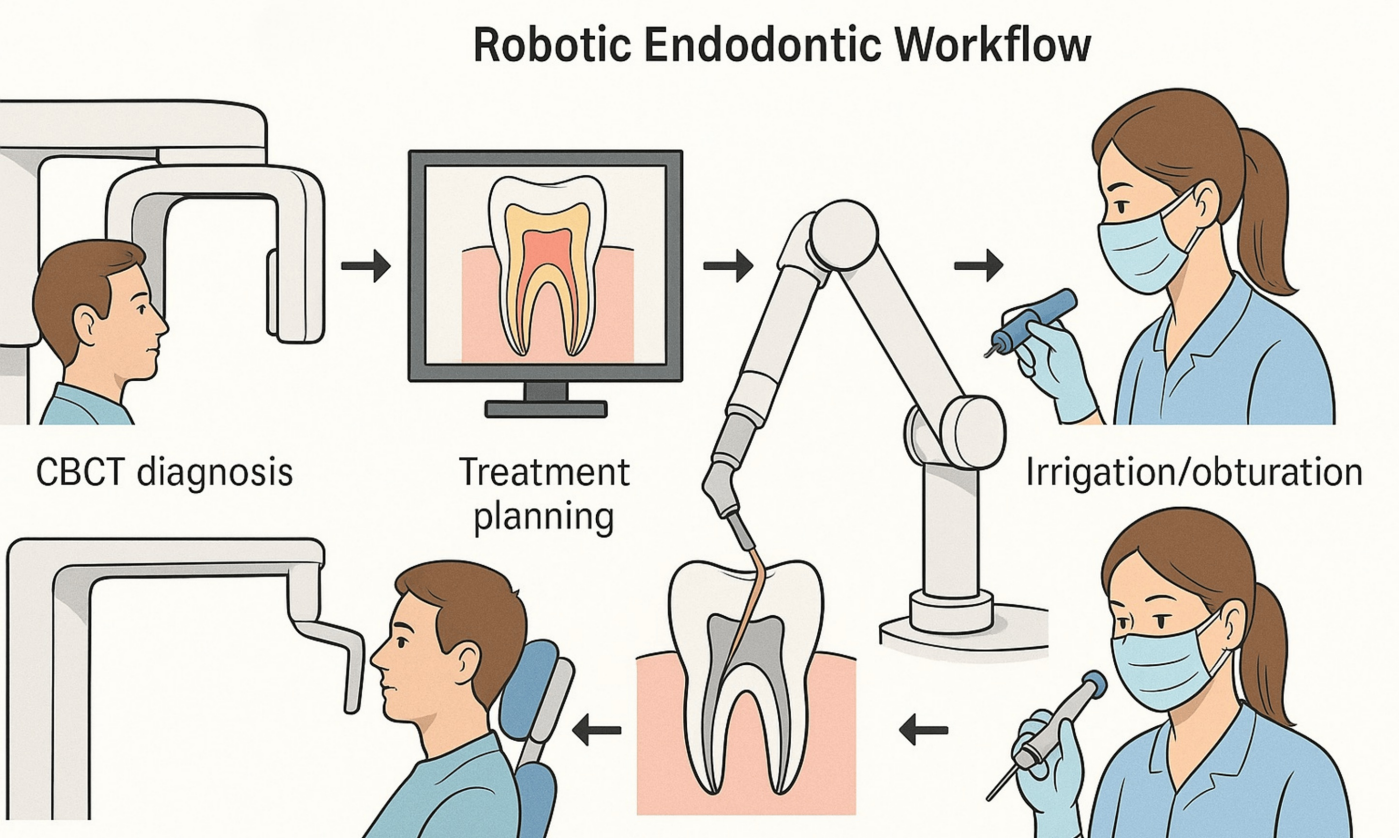
Sequence of robotic procedures in endodontics. Image credit: Rawan S. Alrehaili. CBCT, cone beam computed tomography

Endodontic retreatment poses unique challenges, especially when encountering posts, calcified canals, or obturating materials that obstruct access to the root canal system. Robotic assistance in this area has been suggested conceptually to improve precision and safety during post removal or canal negotiation. However, no peer-reviewed clinical systems are currently available, and existing proposals remain at the level of theoretical development or early experimental exploration. This highlights a promising but as yet unvalidated frontier for endodontic robotics. Moreover, AI-enhanced robotic platforms are being designed to detect the location and extent of previous obturation materials and to assist in their mechanical and chemical removal [26]. This could improve the efficacy of retreatment procedures and reduce reliance on solvent-based techniques that carry additional risks (Figure 2).

Robotics in orthodontics

The application of robotics in orthodontics has evolved from conceptual experimentation to practical integration, redefining the precision and efficiency of orthodontic care. Robotics is now being employed across various domains, including but not limited to aligners, robotic archwire bending, automated bracket placement, image-guided diagnostics, AI-assisted treatment planning, and the development of functional orthopedic and sleep-related appliances. These technologies aim to reduce operator variability, enhance biomechanical control, and optimize clinical workflows, all while offering patients more individualized and efficient care [27].

Robotic Archwire Bending

Orthodontic archwires must be bent and contoured with great accuracy to deliver controlled forces that guide teeth into their desired positions. Conventionally, this process relies on the orthodontist's manual dexterity, which can be inconsistent and prone to fatigue-related errors. The laborious nature of manual bending also limits its reproducibility, especially when dealing with complex treatment mechanics. Robotic wire-bending systems address these limitations by automating the bending process based on 3D treatment simulations, thereby ensuring uniformity and precision in wire fabrication [28,29].

Major Robotic Systems for Wire Bending

SureSmile system (OraMetrix, Inc., Richardson, TX): Developed to streamline fixed appliance therapy, SureSmile employs intraoral scans or CBCT imaging to construct a virtual 3D model of the patient’s dentition. The orthodontist designs a digital treatment plan, which is transmitted to a centralized laboratory where robotic arms equipped with thermal-forming tools bend the archwires. The system’s ability to control wire configuration down to fractions of a millimeter reduces the need for chairside adjustments [30].

Insignia system (Ormco Corporation, Orange, CA): This platform merges digital treatment planning with the robotic fabrication of individualized brackets and archwires. Insignia's software simulates desired tooth movement, and the system fabricates a set of appliances tailored to those movements. Robotic precision ensures accurate force application, which has been correlated with shorter treatment times and improved alignment outcomes [31].

Lingual Archwire Manufacturing and Design Aid (LAMDA) and Bending Art System (BAS): LAMDA was one of the first programmable robots to explore automated wire manipulation using artificial muscle actuators. BAS, on the other hand, relies on servo-controlled multi-joint manipulators to produce customized bends. These systems integrate with orthodontic planning software to execute a series of pre-calculated bends based on anatomical data [32].

Cartesian-based and multi-axis robots: More recent innovations involve Cartesian-type robots that utilize translational and rotational movements to achieve complex spatial wire configurations. Systems with six or more degrees of freedom, such as those based on the Motoman UP6 model, are capable of twisting and shaping wires in multiple planes simultaneously, offering unmatched control over torque and angulation [33].

Comparative Efficiency

Quantitative analyses have shown that robotic archwire systems outperform manual wire bending in several domains. Time efficiency is improved by minimizing the need for repeated adjustments. Additionally, clinical outcomes benefit from greater force consistency, leading to fewer unplanned visits and enhanced predictability. The fatigue life of thermally activated nickel-titanium wires is also preserved when formed under robotic conditions, extending the functional lifespan of the appliances [34,35].

Imaging and Simulation

High-fidelity 3D imaging has become integral to modern orthodontics. Robotic systems have contributed to improving imaging quality by enabling motion-stabilized CBCT acquisition and optimized patient positioning during scans. Robotic chairs and head-positioning units equipped with infrared tracking minimize motion blur and improve reproducibility in serial imaging, which is vital for treatment monitoring and surgical planning [36].

Cephalometric and Morphometric Analysis

AI-assisted robotic platforms have enhanced the process of cephalometric tracing by automatically identifying anatomical landmarks in two-dimensional and 3D datasets. These systems utilize deep learning models trained on large image libraries, which allows for consistent, reproducible measurements. In cases involving skeletal asymmetry or syndromic malocclusions, these tools provide a more objective analysis than traditional manual tracing [37].

Integration with Predictive Algorithms

Robotic planning interfaces are now being merged with machine learning algorithms that can simulate entire treatment pathways. These platforms analyze baseline malocclusion types, predict tooth movement responses, and suggest optimal force systems. This information is directly linked to robotic systems that fabricate appliances according to the AI-generated prescription, thereby closing the loop between diagnosis and appliance production [28].

Robotic Bracket Placement and Bonding

Mechanized bonding platforms: The accuracy of bracket placement plays a pivotal role in the success of orthodontic therapy. Robotic bonding systems, though still in early phases of clinical application, have demonstrated the ability to position brackets on dental models with micrometric precision. These systems operate based on digital setups and eliminate operator-induced variability by maintaining consistent bonding parameters across all teeth [38].

Clinical workflow integration: In an ideal setup, digital scans are imported into a robotic platform that creates a virtual bonding plan. A robotic arm then transfers this plan to either an indirect bonding tray or directly applies the brackets onto the patient's teeth. Feedback-controlled pressure sensors and optical verification modules enhance the accuracy and safety of the procedure [39].

Functional Orthopedics and Sleep Appliances

Robotic simulation of orofacial dynamics: Functional orthopedic appliances aim to modulate growth patterns or guide neuromuscular adaptation. Robotics offers a powerful tool for simulating orofacial dynamics in silico before appliance fabrication. Robotic platforms with actuators that mimic masticatory and lingual movements are being used to evaluate how devices interact with soft tissues and temporomandibular joints (TMJs) [40].

Designing mandibular advancement devices: For patients with obstructive sleep apnea, robotic simulations of mandibular protrusion and airway patency assist in designing customized advancement devices. These systems analyze how changes in mandibular positioning influence pharyngeal airway space, thereby guiding the design of appliances with maximal therapeutic benefit and minimal discomfort [41].

**Figure 3:**
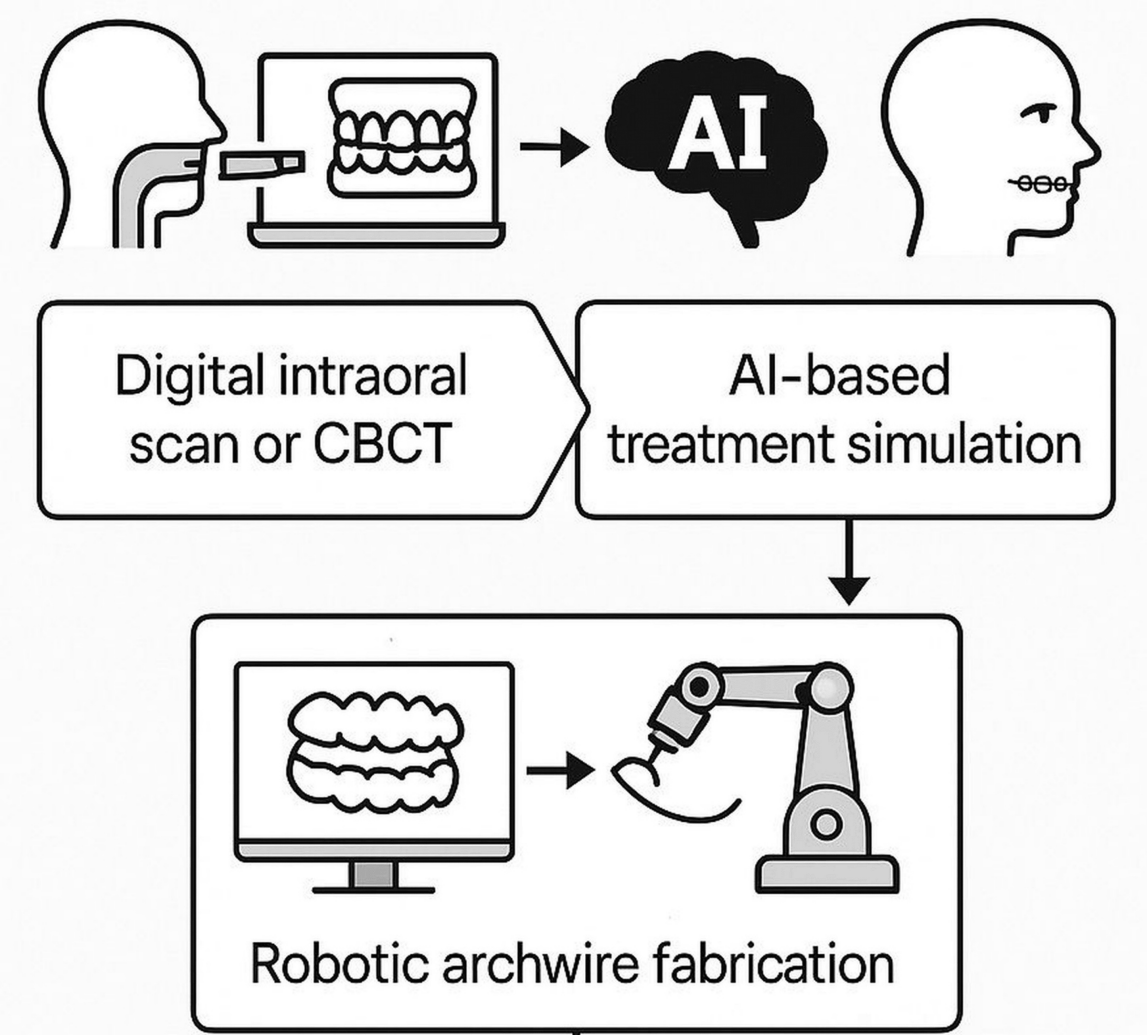
Robotic workflow in orthodontics. Image credit: Rawan S. Alrehaili. CBCT, cone beam computed tomography

Adaptive appliances with real-time feedback: Innovations in sensor-integrated appliances are opening new avenues for smart orthodontics. Robotic platforms are being developed to create appliances that adapt their force output in response to changes in the oral environment, using pressure sensors, gyroscopes, and accelerometers [42]. These appliances could eventually deliver optimized forces that evolve based on patient-specific movement patterns, compliance, and growth (Figure 3).

Robotics in pediatric dentistry

Robotics in pediatric dentistry marks a significant advancement in how dental care is delivered to children, offering greater accuracy, improved safety, and a better overall experience for young patients. By integrating robotic technologies, dental professionals can perform procedures with enhanced control and reliability. This not only helps reduce treatment-related stress for children but also leads to more predictable results and higher standards of care [12].

Behavior Management

Behavior management is a cornerstone of pediatric dentistry, and robotics is playing an increasingly important role in this area. Social robots are being utilized in dental clinics to engage with children, explain procedures in a friendly manner, and provide distraction during treatments. These robots help alleviate anxiety, foster positive attitudes toward dental care, and improve cooperation during visits. By monitoring physiological signals such as heart rate or facial expressions, robotics can also provide real-time feedback to clinicians, allowing for tailored behavioral interventions that further enhance the patient experience [43].

Robotic-Assisted Dental Procedures

Robotic-assisted dental procedures are redefining pediatric dental care by providing enhanced precision, dexterity, and control during surgical interventions. These systems facilitate minimally invasive surgeries, such as the extraction of supernumerary teeth or cyst removal, which is particularly advantageous for children due to their smaller oral structures and heightened sensitivity. The use of robotics minimizes tissue trauma, shortens recovery times, and improves overall procedural outcomes. Additionally, robotic arms can offer steady, repeatable movements that reduce the risk of human error, ensuring safer and more predictable results for young patients [44].

Imaging and Diagnostics

The incorporation of robotics into dental imaging and diagnostics has significantly improved the quality and safety of radiographic procedures in pediatric dentistry. Robotic arms are capable of positioning radiographic equipment with exceptional accuracy, which minimizes the need for repeat exposures and ensures optimal image quality. This is especially important for children, as it helps reduce their radiation exposure. Furthermore, robotics can aid in the early detection of dental anomalies, caries, and developmental issues by enabling more consistent and reproducible imaging techniques [45].

Guided Intraoral Scanning 

Robotic-guided intraoral scanning has revolutionized the process of obtaining digital impressions in pediatric patients. These systems provide highly accurate scans, which are critical for the fabrication of restorations, crowns, and orthodontic appliances. The precision of robotic scanning eliminates the discomfort often associated with traditional impression materials and reduces the likelihood of errors that could necessitate repeat appointments. This technology not only enhances the fit and function of dental appliances but also streamlines the workflow for both clinicians and patients [45].

Training and Simulation

Robotic simulators are transforming the education and training of pediatric dentists. These advanced systems create highly realistic practice environments where practitioners can refine their skills on virtual pediatric patients before treating real ones. Features such as haptic feedback simulate the tactile sensations of dental procedures, helping trainees develop the delicate touch required for pediatric care. This technology not only boosts clinical competence and confidence but also contributes to safer and more effective patient care [46].

Automation in Dental Laboratories

The automation of dental laboratory processes through robotics has brought about significant improvements in the fabrication of pediatric dental prosthetics, crowns, and orthodontic devices. Robotic systems ensure high levels of accuracy and consistency, resulting in better-fitting appliances and reduced turnaround times. Additionally, automated quality control by robotics helps maintain stringent standards, minimizing the risk of defects in dental products used for children. This level of precision and efficiency is essential for meeting the unique needs of pediatric patients [1].

Robotics in implant dentistry

The landscape of implant dentistry has rapidly evolved with the integration of robotic systems, transforming it from a highly technique-sensitive practice to a more standardized and precision-driven discipline. Robotic-assisted implantology promises increased accuracy, reduced surgical trauma, shorter recovery times, and reproducibility across operators. These benefits are particularly crucial in anatomically challenging cases, such as those involving proximity to the inferior alveolar nerve or maxillary sinus [47].

Robotic Systems Functionality

Robotic systems in dental implant surgery are typically categorized by their level of autonomy. Active systems are designed to perform procedures independently, following a preoperative plan while the surgeon supervises without directly guiding the instrument in real time. Passive systems, by contrast, require the operator to manually control the robotic arm while receiving visual or haptic feedback that enhances precision. Between these extremes are semi-active systems, which represent a hybrid model: the robot executes specific procedural steps, but manual interaction is still required during entry, exit, or real-time adjustments [48].

Technological Infrastructure of Implant Robots

Most robotic systems consist of a mechanical arm for drilling or implant insertion, a visual tracking or coordinate measurement machine (CMM) to guide motion, and a control unit with integrated software that often incorporates real-time imaging and preoperative planning tools. The robot may either be anchored to a fixed base or be mobile. Real-time feedback - both visual and tactile - ensures responsive and adaptive performance, allowing the operator to intervene if necessary [49].

Robotic Systems in Implant Dentistry

Yomi (Neocis, Inc., Miami, FL): The Yomi system, cleared by the U.S. Food and Drug Administration (FDA) in 2017, is a passive robotic platform that assists surgeons with drilling and implant placement. It uses a CMM-based robotic arm that provides real-time visual and physical guidance while allowing the clinician to control the procedure manually. Despite its passive classification, Yomi offers enhanced safety and precision compared to freehand techniques [50].

Remebot (Beijing Baihui Weikang Technology Co., Ltd., Beijing, China) is a semi-active robotic system developed in China. It features dual robotic arms: one for positioning and the other for drilling and implant placement. Using preoperative CBCT data, it can autonomously prepare the implant site and assist with placement under the surgeon’s supervision. It combines autonomy with tactile feedback and foot-controlled adjustments [51].

YekeBot (Beijing Institute of Robotics and Intelligent Systems, Beijing, China) is a fully autonomous implant robot designed to perform implant surgeries independently, including drilling, implant insertion, and intraoral positioning. The robot follows pre-set coordinates but allows surgeon oversight for safety checks and procedural verification [52].

Theta and DentRobot: Theta (Beijing Fulei Medical Technology Co., Ltd., Beijing, China) and DentRobot (Beijing Tianzhi Robotics Co., Ltd., Beijing, China) are more recent innovations that offer semi-active and passive modalities, respectively, supporting procedures with optical tracking and foot-pedal control for intraoral movements. These systems emphasize real-time responsiveness and operator control, even while performing guided functions [50].

Clinical Efficacy and Time Efficiency

Clinical trials and preclinical studies have shown that robotic systems can improve the precision of implant positioning and reduce the likelihood of neurovascular complications [53]. In addition to accuracy, these systems have been shown to increase procedural efficiency by reducing intraoperative adjustments and minimizing deviations from the planned surgical pathway.

Advantages Over Conventional Techniques

When compared with traditional freehand or guided methods, robotic platforms offer several important benefits. Their ability to deliver submillimeter accuracy is particularly valuable in flapless implant surgery and in anatomically sensitive regions where the margin for error is minimal [54]. By relying on standardized, software-driven protocols, robotic systems also help to reduce human error and mitigate the variability that arises from differences in operator skill or fatigue [55]. Another advantage lies in their role in dental education, where robotic simulation platforms provide opportunities for training in complex surgical scenarios. These educational tools allow practitioners to refine skills in a controlled environment, ultimately enhancing competence and confidence before procedures are performed on patients [56].

Robotics in OMFS

The integration of robotics into OMFS marks a transformative shift in how complex surgical interventions are conceptualized and performed. Robotics in OMFS enhances visualization, surgical precision, access to anatomically restricted areas, and operator ergonomics. Although robotic technology has been well-established in general surgery and urology, its adoption in maxillofacial procedures has gained traction in recent years, particularly in orthognathic surgery, tumor resection, microvascular reconstruction, and TMJ interventions [57].

*Early Adaptations in Head and Neck Surger*y

The earliest applications of surgical robotics in the craniofacial domain were seen in transoral robotic surgery (TORS) for oropharyngeal cancers. These procedures utilized the da Vinci Surgical System to access tumors via the oral cavity without external incisions, thus minimizing morbidity. The precision and control demonstrated in TORS paved the way for robotic adaptation in more intricate skeletal surgeries [58].

Transition to Hard Tissue Procedures

Initial concerns about robotic applicability in bony procedures, such as limited bone-cutting capabilities, were gradually addressed through integration with computer-aided design and manufacturing (CAD/CAM) and dynamic navigation systems. This shift enabled the use of robots in tasks like osteotomies and precise fixation of bone segments in orthognathic surgery [59].

Robotic Systems Used in OMFS

Robotic systems applied in OMFS are often borrowed from other surgical disciplines and adapted for craniofacial procedures. The da Vinci Surgical System is primarily used for soft tissue interventions, including TORS, tongue base reduction, and tumor excision. Orthopedic systems such as ROBODOC (Curexo Technology Corporation, Fremont, CA) and MAKO (Stryker Corporation, Kalamazoo, MI) have also been applied to perform high-precision osteotomies in jaw surgeries. More recently, customized robotic arms have been designed specifically for maxillofacial applications, incorporating haptic feedback and intraoperative imaging to enhance surgical control and improve accuracy [9].

Applications in OMFS

Orthognathic surgery: Robotics facilitates enhanced control during mandibular and maxillary osteotomies, improving symmetry and precision. When integrated with virtual surgical planning (VSP) and 3D-printed guides, robots can translate digital surgical plans into real-time execution with submillimeter accuracy [60].

Tumor resection and ablative surgery: Robotic-assisted resections in anatomically constrained areas, such as the base of the tongue or retromolar trigone, offer improved visualization and tissue preservation. The enhanced dexterity of robotic instruments allows surgeons to operate in regions that are otherwise difficult to access, reducing the need for extensive external incisions. This minimally invasive approach not only shortens recovery time but also lowers perioperative morbidity. As a result, patients often experience better functional outcomes, particularly in terms of speech and swallowing, when compared with conventional open surgical methods [61].

Microvascular reconstruction: Precision in flap harvest and vascular anastomosis is critical in reconstructive OMFS. Robotic platforms, with motion scaling and tremor filtration, aid in microsurgical tasks by offering superior stability and access to deep surgical fields. These capabilities enhance the surgeon’s ability to achieve fine suturing and vessel approximation, which are often technically demanding under conventional conditions. In addition, robotic assistance can reduce surgeon fatigue during prolonged microsurgical procedures, thereby improving both accuracy and overall surgical outcomes [62].

TMJ interventions: Robotic assistance in TMJ arthroplasty or arthroscopy allows for precise placement of prosthetic components and real-time navigation in a highly mobile and anatomically sensitive joint. These procedures benefit from the improved dexterity and limited invasiveness that robotics provides [63].

Integration with digital planning and intraoperative navigation: The synergy between robotic surgery, virtual surgical planning, and intraoperative navigation systems represents a pivotal advancement. Preoperative 3D models derived from CT or MRI data enable virtual rehearsal, which is then translated into robotic execution. Intraoperative navigation ensures that deviations from the planned trajectory are immediately corrected, enhancing procedural accuracy and safety [64].

Training, simulation, and skill acquisition: Robotic platforms in OMFS are increasingly being utilized in simulation environments for resident education. Haptic-enabled simulators and augmented reality interfaces provide trainees with realistic, risk-free surgical practice. Moreover, robotic systems allow for quantitative assessment of surgical performance through motion tracking and force application metrics [65].

Limitations and challenges

Despite the rapid advancement and promising potential of robotics in dental specialties, several significant limitations and barriers continue to constrain its widespread clinical adoption. These challenges span technological, clinical, economic, educational, and regulatory domains, each influencing the feasibility and practicality of robotic integration into routine dental practice.

Technological and Infrastructural Barriers

The deployment of robotic systems in dentistry requires a highly specialized and robust technological infrastructure. Many systems necessitate the integration of advanced imaging modalities such as CBCT, intraoral scanners, and motion capture systems, along with compatible treatment planning software and hardware interfaces. Synchronizing these components demands not only a high initial investment but also continuous technical maintenance and calibration. Moreover, the miniaturization required for intraoral robotic applications remains a significant engineering challenge. Unlike other surgical disciplines with expansive operative fields, dentistry involves confined anatomical spaces, complex geometries, and dynamic environments. Designing robotic tools that can perform with millimeter precision in such environments while maintaining safety and responsiveness is a critical hurdle yet to be fully overcome.

High Costs and Economic Sustainability

One of the most prominent limitations is the considerable cost associated with acquiring, installing, and maintaining robotic systems. These costs encompass not only the base price of the robotic units themselves but also the software licensing fees, technical support, consumables, and frequent system upgrades. In many cases, centralized fabrication models (e.g., SureSmile or Insignia) involve recurring service contracts and turnaround delays. For private dental practices, especially those operating in resource-constrained settings, the return on investment (ROI) for robotic systems is often difficult to justify unless the system is used at scale or contributes directly to enhanced case throughput or premium services. Until the costs decrease and reimbursement models adapt to recognize robotic-assisted procedures, the financial barrier will remain a limiting factor for broader accessibility.

Clinical Workflow Integration and Time Efficiency

While robotic systems promise enhanced precision and reproducibility, their integration into existing clinical workflows is not always seamless. Robotic procedures often require additional imaging, data preparation, and software planning steps before execution. These pre-treatment processes may offset the time savings achieved during the clinical procedure itself. Moreover, real-time adaptability during procedures remains limited. Unlike human clinicians who can respond dynamically to intraoperative variations, robotic systems, especially semi-autonomous ones, are often constrained by pre-programmed instructions and lack tactile intuition. The absence of haptic perception in many robotic tools can reduce their effectiveness in scenarios requiring nuanced decision-making, such as negotiating calcified canals or responding to unexpected bleeding during surgery.

Learning Curve and Operator Training

The implementation of robotics requires practitioners to acquire a new set of technical skills that extend beyond traditional dental education. These include competencies in digital imaging, treatment planning software, hardware calibration, and real-time system management. For many clinicians, especially those with limited exposure to digital dentistry, the learning curve may be steep. Moreover, the variability in robotic platforms means that training is often vendor-specific, lacking a standardized educational framework across systems. This lack of uniform training may compromise consistency, limit cross-platform operability, and slow down the integration of robotics into mainstream clinical curricula.

Ethical, Legal, and Regulatory Concerns

The rise of robotic and AI-driven dentistry introduces new layers of ethical and legal considerations. Questions surrounding liability, particularly in the event of system errors, software malfunctions, or adverse outcomes, remain unresolved. It is often unclear whether responsibility lies with the practitioner, the manufacturer, or the software designer, especially when decision-making is shared between human and machine. Furthermore, regulatory agencies have yet to establish comprehensive frameworks for evaluating and approving robotic systems tailored for dental use. The absence of standardized guidelines can delay product development, discourage investment, and create uncertainty for early adopters. Data privacy and cybersecurity, especially in cloud-based systems, also pose challenges given the sensitive nature of patient health records and diagnostic data.

Patient Acceptance and Trust

Although many patients may perceive robotic interventions as innovative and high-tech, others may be apprehensive about being treated by a machine. Concerns about safety, depersonalization of care, and the potential for mechanical errors may reduce patient acceptance, particularly among older individuals or those unfamiliar with digital healthcare. Building patient trust in robotic systems requires transparent communication, clinician endorsement, and demonstrated benefits in terms of outcomes, comfort, and efficiency. Until robotic dentistry becomes more routine, some resistance based on fear or skepticism is likely to persist. A summary of the key benefits and associated challenges is presented in Table 2.

**Table 2:** Benefits and challenges of robotics integration.

Domain	Benefits	Challenges
Clinical Outcomes	Increased precision, reproducibility	High initial investment and maintenance costs
Patient Experience	Reduced chair time, minimally invasive procedures	Limited patient familiarity and trust
Training and Education	Objective feedback, skill standardization	Requires new curricula and instructor retraining
Workflow Efficiency	Standardized protocols, AI integration	Integration with existing infrastructure is complex
Research and Data	Digital treatment logs, measurable outcomes	Ethical and legal concerns around autonomy and AI use

Future directions and perspectives

The trajectory of robotics in dentistry is shaped by continuous innovation in AI, mechatronics, imaging technologies, and biomedical engineering. As these domains converge, the next generation of robotic systems is expected to be smarter, more adaptable, and better integrated into clinical workflows. The following emerging directions highlight key areas of development that will likely define the future landscape of dental robotics.

Miniaturization and Enhanced Dexterity

One of the foremost goals in dental robotics is further miniaturization of robotic instruments to enable safer, more accurate performance in the confined oral environment. Advances in soft robotics, nanomaterials, and microfabrication are driving the development of intraoral robots with greater flexibility and biomechanical compatibility. These innovations may allow robotic arms or microrobots to navigate hard-to-reach anatomical regions such as curved root canals, deep periodontal pockets, or subperiosteal implant sites with minimal iatrogenic risk. In tandem, improvements in end-effectors, sensor integration, and actuation mechanisms will enhance the dexterity and responsiveness of robotic tools. This will allow not only greater precision in surgical tasks but also adaptability to intraoperative changes in real time.

Integration of AI and Machine Learning

The incorporation of AI into dental robotics is poised to shift these systems from passive executors of pre-programmed commands to intelligent collaborators capable of autonomous reasoning. Machine learning algorithms can analyze vast amounts of patient data, including imaging, clinical history, and biomechanical parameters, to generate optimized treatment plans tailored to individual cases. In real-time applications, AI-enabled robotics could perform dynamic risk assessment, adjust force vectors during procedures, or even halt operations if unexpected complications are detected. In orthodontics, AI may predict tooth movement patterns, while in implantology, AI may aid in selecting optimal implant sites based on bone density and occlusal loading patterns.

Real-Time Imaging and Feedback-Controlled Robotics

Future dental robots will likely incorporate real-time imaging modalities, such as high-definition intraoral cameras, OCT, and even augmented reality overlays, to guide procedures with continuous visual and positional feedback. These systems will enable closed-loop control, where intraoperative data influence robotic behavior moment-to-moment, allowing for automated corrections in trajectory, speed, and depth of action. Such feedback-rich environments will be particularly valuable in procedures requiring high spatial sensitivity, such as endodontic microsurgery or sinus floor elevation, where proximity to critical structures is a constant concern.

Expanded Role in Education and Remote Dentistry

As robotic simulation becomes more sophisticated, its role in dental education will expand beyond preclinical training into postgraduate and continuing professional development. Robotic haptic trainers, coupled with performance analytics, will provide individualized feedback and allow for the refinement of complex motor skills under various simulated clinical conditions. Moreover, with increasing global emphasis on telemedicine, the possibility of remote-controlled dental robotics is under exploration. This could enable experienced clinicians to assist or supervise procedures in underserved or rural areas, dramatically expanding access to specialized care.

Personalized and Predictive Treatment Models

The future of robotic dentistry will be closely aligned with the vision of personalized medicine. Using patient-specific data from genetic profiling, bone metabolism markers, and 3D anatomical modeling, robotic systems will contribute to designing treatments that optimize biological compatibility and long-term success. Predictive analytics may also enable early detection of pathological changes through robotic diagnostic tools embedded in daily-use dental devices, allowing for proactive and preventive intervention strategies.

Regulatory Harmonization and Clinical Validation

**Figure 4:**
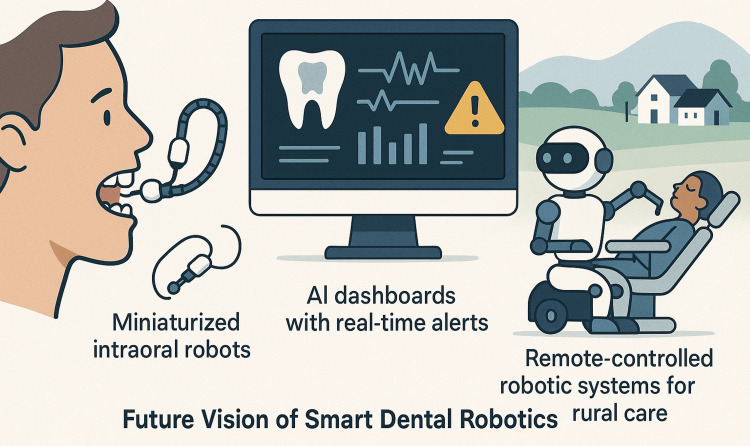
Conceptual landscape of smart dental robotics. Image credit: Rawan S. Alrehaili.

For robotics to become a mainstay in dental practice, robust clinical trials and multicenter validation studies will be essential to confirm efficacy, safety, and cost-effectiveness. Parallel to this, regulatory agencies must establish unified frameworks that address device classification, liability, and quality control. Future systems will benefit from clearly defined guidelines and pathways for approval, fostering innovation while safeguarding patient safety. The incorporation of robotics into clinical guidelines, dental school curricula, and evidence-based protocols will also help shift perception from novelty to necessity. As visualized in Figure 4, the future of dental robotics is likely to involve AI integration, miniaturization, and remote operability.

## Conclusions

Robotics is being explored as a tool that could expand the possibilities of dental care and reshape aspects of modern dentistry by enhancing precision, safety, and treatment efficiency. Its integration may help reduce human error and procedural variability while supporting greater customization, standardization, and data-driven decision-making. Although challenges such as cost, training, and regulatory oversight remain, ongoing research and development suggest that robotics has the potential to become an important component of the dental armamentarium. Rather than being seen solely as a technological enhancement, dental robotics may represent a step toward elevating standards of care, broadening access to complex procedures, and advancing digitally guided, patient-centered precision dentistry.
